# Construction of a pathway-independent three-module self-induced regulatory system and its application in 3-hydroxypropionic acid production

**DOI:** 10.1016/j.engmic.2026.100281

**Published:** 2026-06-05

**Authors:** Yaping Gao, Wendi Xu, Xiaoya Yang, Fei Gu, Sumeng Wang, Qingsheng Qi, Quanfeng Liang

**Affiliations:** a State Key Laboratory of Microbial Technology, Shandong University, Qingdao 266237, China; b Qingdao Agricultural University, Qingdao 266100, China

**Keywords:** Multimodule self-induced regulatory system, Pathway-independent, Temporal regulation, Metabolic engineering

## Abstract

Dynamic regulatory systems are promising tools for the biosynthesis of high-value chemicals. However, the construction of pathway-independent multimodule self-induced regulatory systems capable of modulating complex metabolic pathways remains challenging. In this study, we developed a multimodule self-induced regulatory system based on two orthogonal quorum sensing (QS) and a stationary-phase sensing system. We optimized the Las and Tra* QS response times by tuning acyl-homoserine lactone (AHL) synthase expression, identified a stationary-phase sensor with a higher output, and integrated these into a pathway-independent multimodule self-induced regulatory system. This system successfully enabled the sequential expression of multiple genes at specified time intervals under autoinduction conditions. Ultimately, this system, combined with a small RNA inhibition module, was employed to produce 3-hydroxypropionic acid (3-HP), which sequentially regulated the three modules of the tricarboxylic acid cycle, fatty acid metabolism, and 3-HP synthesis. Application of this system resulted in a 3-HP titer of 7.0 g/L in shake flask fermentation, which was 27% higher than that of the base strain using a static regulatory strategy, while also improving bacterial growth. This application validates the potential of the developed multimodule self-induced regulatory system to regulate multigene expression in complex microbial metabolic networks.

## Highlight

• Constructed a three-module self-induced regulatory system.
• The multimodule self-induced regulatory system is independent of metabolic pathways.
• Temporal regulation of multiple gene expressions.
• The application potential has been demonstrated for the production of 3-HP.

## Graphical abstract

**Figure:**
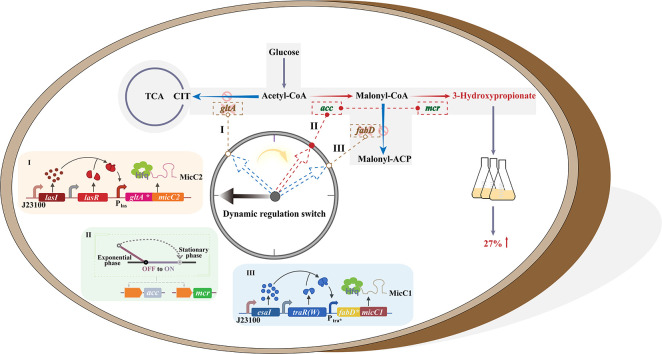

## 1 Introduction

Various metabolic engineering strategies are used to regulate the expression of genes related to microbial metabolism [[1], [2], [3]]. Traditional static regulatory strategies often impose a substantial metabolic burden on the host cells, resulting in an imbalance between cellular growth and target product synthesis. Dynamic regulation has proven to be an effective approach for constructing efficient microbial cell factories. For instance, Farmer et al. first demonstrated the efficacy of dynamic regulation strategies for enhancing lycopene titers [4]. Dynamic regulation of the phosphoenolpyruvate metabolic node in *Escherichia coli* enables the autonomous partitioning of carbon flux between the bacterium’s native metabolic pathways and heterologously engineered muconic acid biosynthetic route, consequently boosting muconic acid yields [5]. Implementation of dynamic regulatory strategies to allocate the carbon flux between cellular growth-essential and heterologous pathways for high-value chemical production minimizes resource consumption for non-essential cellular processes, mitigates the accumulation of toxic intermediates in cells, and improves the overall fermentation productivity [[6], [7], [8]]. Consequently, dynamic regulation strategies have attracted considerable attention in recent years [9].

The most commonly employed method in metabolic engineering is single-module regulation, which is relatively straightforward [[10], [11], [12]]. However, the complexity of metabolic pathways and cellular regulation in biosynthesis often renders single-module regulation inadequate [13]. Consequently, multimodule regulation strategies have been developed to simultaneously regulate various metabolic pathways or the expression of multiple genes, thereby enhancing the yield and titer of the target products [14]. As shown in Table 1, single- and double-module regulation strategies are currently the most prevalent hierarchical strategies employed in production. However, despite the significant theoretical advantages of multimodule regulation strategies, their practical development remains highly challenging, leading to a fragmented research landscape and limited universality in this field. The few existing three-module systems are heavily constrained by the design logic, which is highly specific to particular biosynthesis contexts. In a study by Zhou et al. [15], the naringenin synthesis pathway is the first regulation module, while a dynamic regulatory network responding to naringenin levels is the second module. As naringenin concentration increases, the fatty acid module is progressively inhibited. The third regulation module employs a p-coumaric acid biosensor that detects the accumulation of p-coumaric acid in cells, thereby enhancing the synthetic pathway of the precursor malonyl-CoA. The integration of these three modules into the metabolic regulation network, which is responsive to specific metabolites, significantly enhances the efficiency of naringenin production. Liu et al. specifically designed a three-module engineered strain to treat salicylic acid contaminants in industrial wastewater [16]. The first module employs salicylic acid as an inducer to activate the salicylic acid sensor for detection. The second module expresses salicylate 5-hydroxylase using a stationary phase promoter to facilitate the degradation of salicylic acid. The third module utilizes a salicylic acid-responsive promoter to express a suicide circuit, enabling the strain to die after salicylic acid degradation. The work mentioned above validates the use of multimodule regulation for the dynamic regulation of multiple metabolic fluxes. However, these three-module regulatory systems are responsive to metabolites from specific pathways, which significantly limits their applicability. The development of a self-induced, universal, pathway-independent, three-module regulatory system remains a critical challenge.

**Table 1:** Summary of hierarchical dynamic regulation strategies with different numbers of regulatory modules.

Number of Regulatory module	Sensing Signal	Pathway Dependency	Reference
1	Lysine	Yes	[17]
1	cell density	No	[18]
1	Pyruvate	Yes	[19]
1	Cumate	No	[20]
1	cell density	No	[21]
2	vanillin, ferulic acid	Yes	[22]
2	myo-inositol, cell density	Yes	[23]
2	Temperature, pyruvate	Yes	[24]
2	cell density	No	[25]
3	naringenin, p-coumaric acid	Yes	[15]
3	stationary-phase, salicylic	Yes	[16]
3	Cell density, stationary phase	No	This study

Quorum sensing (QS)-based dynamic regulation is a pathway-independent method for modulating cellular metabolism in response to cell density [26]. As natural microbial communication systems, QS system can respond to the levels of local cell populations and regulate various cellular physiological behaviors [[27], [28], [29], [30]]. As synthetic biology and metabolic engineering continue to advance, QS systems have found broad utility in designing gene circuits, constructing synthetic biological systems, and producing target metabolites [31]. Gupta et al. applied the QS system to dynamically downregulate the expression of phosphofructokinase 1, thereby facilitating a greater flow of glucose-6-phosphate into the production pathway and enhancing myoinositol production [32]. In our previous study, two completely orthogonal QS systems with self-produced auto-inducers were built to enable the systems to be signal orthogonal and promoter orthogonal to each other. The systems were applied intracellularly, and an automatic delayed cascade circuit was successfully demonstrated, which could realize sequential gene expression without an exogenous inducer [33]. The QS system is extensively used in metabolic engineering because of its independence from metabolic pathways, allowing dynamic regulation of gene expression to improve production [34].

In this study, we aimed to construct a self-induced universal three-module regulatory system to achieve the multimodule-coordinated regulation of biosynthetic pathways. Self-induction refers to a regulatory process triggered by intrinsic cellular signals, such as cell density and growth phase, without the need for exogenous inducers. This system was designed based on cellular physiological state signals rather than specific metabolite concentrations, making its activation mechanism independent of pathway metabolites; hence, we termed it a pathway‑independent three‑module self‑induced system. To build this system, we employed orthogonal Las and Tra* QS systems together with an orthogonal stationary-phase sensing system and then combined them with a small RNA inhibition module for the biosynthesis of 3-hydroxypropionic acid to evaluate its application potential. Compared with existing dynamic regulation schemes, this three-module regulatory system represents a significant shift in design philosophy, moving from the customized optimization of individual metabolic pathways to the establishment of a universal regulatory framework adaptable to diverse biosynthetic routes. This design enables the engineering of strains for new target products without the need to reconstruct the dynamic regulatory networks from scratch. By simply replacing the corresponding biosynthetic pathway modules, strains with autonomous and temporally regulated capabilities can be rapidly obtained. This approach enhances the universality and construction efficiency of dynamic regulatory strategies in metabolic engineering, demonstrating important application prospects.

## 2 Materials and methods

### 2.1 Strains, plasmids, primers, and culture medium

The strains, plasmids, and primers used in this study are listed in Supplementary Tables S1, S2, and S3, respectively. *E. coli* DH5α was used for plasmid construction, and *E. coli* TOP 10 served as the host strain for protein expression and fermentation. Primers for plasmid construction were synthesized using polymerase chain reaction (PCR) and Gibson assembly [35].

The main plasmids used in our study were pTra* and pLas, constructed by Jiang et al. [33]. The J23107 and P_tra*_ fragments were assembled upstream of *esaI* and *gfp*, respectively. Using pTra* as the template, the primers Tra-F and Tra-R were employed to amplify the linear backbone fragment, and the primers 107-ESAI-F and 107-ESAI-R were used to obtain the *esaI* sequence under the control of the promoter J23107. Subsequent Gibson assembly of the *esaI* fragment yielded the pTra*−107 plasmid. Additional promoters (J23108, J23100, and J23114; http://parts.iGEM.org/, accessed March 13, 2023) were incorporated into the primer design and assembled into the corresponding *Dpn*I-digested DNA backbones, resulting in the construction of pTra*−108, pTra*−100, and pTra*−114, respectively. Similarly, J23107 and P_las_ fragments were assembled upstream of *lasI* and *gfp*, respectively. Using pLas as the template, the primers Las-F and Las-R were used to generate the linear backbone fragment, whereas the primers 107-LASI-F and 107-LASI-R were used to amplify the *lasI* sequence driven by the promoter J23107. Gibson assembly of *lasI* produced the pLas-107 plasmid, and the incorporation of the J23100 and J23114 promoters into custom primers enabled the construction of pLas-100 and pLas-114, respectively. Promoters P_1.1_, P_2.1_, and P_3.1_ were included in the primers and assembled upstream of *gfp* via Gibson assembly to generate the plasmids pP-1.1, pP-2.1, and pP-3.1, respectively. The *acetyl-CoA carboxylase* (*acc*) and *malonyl-CoA reductase* (*mcr*) genes were PCRamplified from the genomic DNA of *Corynebacterium glutamicum* and *Chloroflexus aurantiacus*, respectively.

To construct a self-induced regulatory strain, the moderate-strength stationary-phase promoter P_3.1_ was selected to drive the expression of *acc* and *mcr*, yielding plasmids pH-II and pL-II. To construct a static control strain (strain 1) with comparable expression levels, we selected constitutive promoters of medium strength from the iGEM website. Promoter J23107 was used to drive *acc* expression, and promoter J23104 was used to drive *mcr* expression, resulting in plasmids pH-I and pL-I, respectively. For the regulation of *gltA* and *fabD* expression, plasmids pH-III and pL-III were generated, respectively. The genetic elements used in strains 1–5 are summarized as follows: strain 1 contained the plasmids pH-I and pL-I, strain 2 contained the plasmids pH-II and pL-II, strain 3 contained the plasmids pH-III and pL-II, strain 4 contained the plasmids pH-II and pL-III, and strain 5 contained the plasmids pH-III and pL-III. Detailed information on the plasmids, promoters, and sequences is provided in Supplementary Tables S2, S4, and S5, respectively.

### 2.2 Characterization of the three-module self-induced system

For fluorescence measurements, *E. coli* TOP10 cells carrying the relevant plasmid constructs were processed as follows. Single colonies were selected and grown overnight in Luria-Bertani (LB) medium broth supplemented with the corresponding antibiotics at 37 °C with shaking at 220 rpm. A 2% (v/v) aliquot of this pre-culture was then added to each well of a 24-well plate, with a final volume of 1 mL per well. No inducers were added throughout the process. Cultivation was carried out at 37 °C with shaking at 220 rpm [36]. The cell density (optical density at 600 nm, OD_600_) and green fluorescent intensity (excitation, 485 nm; emission, 528 nm) were quantified using a Synergy HT Multi-Detection Microplate Reader (BioTek, Winooski, VT, USA).

### 2.3 3-HP fermentation

The M9 medium was used for 3-HP fermentation, comprising 0.15 mg/L NaMoO_4_·2H_2_O, 0.25 mg/L CuSO_4_·5H_2_O, 0.3 mg/L ZnSO_4_·7H_2_O, 0.5 g/L NaCl, 1 g/L NH_4_Cl, 0.7 mg/L CoCl_2_·6H_2_O, 1.25 mg/L H_3_BO_3_, 2.5 g/L glucose, 2 g/L MOPS, 5 g/L yeast extract, 3 g/L KH_2_PO_4_, 6 g/L Na_2_HPO_4_, 246.5 mg/L MgSO_4_·7H_2_O, 2 mg/L vitamin B1 (thiamine) 14.7 mg/L CaCl_2_·H_2_O, 1.6 mg/L MnCl_2_·4H_2_O, and 20 g/L glycerol [36]. The fermentation procedure was carried out as follows: a single colony was first inoculated into LB medium and cultured at 37 °C for 12 h to prepare the primary seed culture. This seed was then transferred with a 1% (v/v) inoculum into the fermentation medium and cultivated for 72 h at 37 °C with shaking at 220 rpm. Fermentation was performed in a 300-mL baffled flask containing 50 mL M9 medium.

### 2.4 Analytical methods

Fermentation samples were diluted, and the cell biomass was determined by measuring the OD_600_ using a UV–Vis spectrophotometer (Shimadzu, City, Japan). For glucose quantification, samples were centrifuged to collect the supernatant, which was then filtered through a 0.22-μm aqueous membrane and analyzed via high-performance liquid chromatography (HPLC, Shimadzu). Product titer was determined using an HPLC system equipped with a diode array detector, where the mobile phase was 0.5 mM H_2_SO_4_ [37]. The final yield was quantified using a standard curve generated from the corresponding reference standards.

## 3 Results and discussion

### 3.1 Construction and optimization of QS systems

**Fig. 1:**
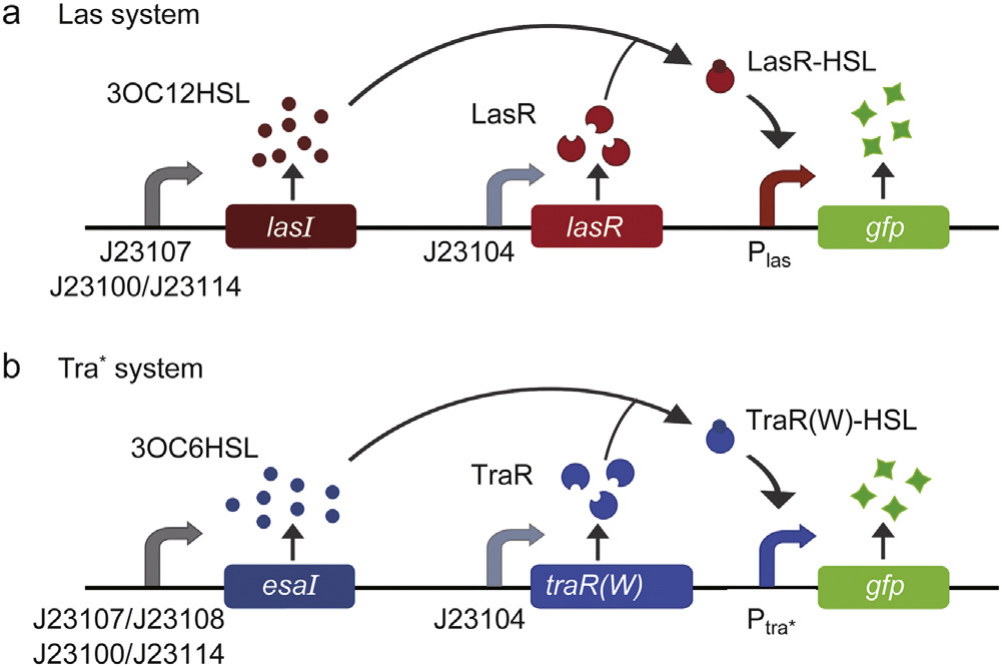
Design of the Tra* and Las quorum sensing (QS) systems. (A) In the Las QS system, LasI synthesizes and secretes the auto-inducer 3OC12HSL. When the concentration of this signaling molecule reaches a certain threshold, it binds to the receptor protein expressed by LasR, resulting in the formation of the complex LasR–HSL. This complex can subsequently activate the P_las_ promoter, which in turn initiates green fluorescent protein (GFP) expression. (B) In the Tra* QS system, the 3OC6HSL synthesized and secreted by EsaI interacts with the TraR(W) receptor protein. The resulting TraR(W)-HSL complex can activate the P_tra*_ promoter, leading to GFP expression. The expression of the TraR(W) and LasR receptor proteins is regulated by the BBa_J23104 promoter.

We selected the Las and Tra* QS systems, both of which exhibit favorable orthogonality based on prior laboratory studies, to design a QS system with expression time intervals [33]. These systems were fused with green fluorescent protein (GFP) and characterized in *E. coli*. Acylhomoserine lactone synthases (EsaI and LasI) continuously synthesize and secrete HSL signals during bacterial growth. When the concentration of HSL reaches a specific threshold, the signal binds to the first receptor protein, TraR(W) or LasR. This binding results in the formation of TraR(W)-HSL or LasR-HSL complexes, which subsequently activate the P_tra*_ or P_las_ promoters, respectively. Once activated, this system induces GFP expression. Notably, the expression intensity of AHL synthase influences the time required for signal molecules to reach the response threshold, thereby affecting the activation timing of the QS system. We further optimized the expression intensity of AHL synthase to obtain Tra* QS and Las QS systems with varying turn-on times. We utilized promoters J23107, J23108, J23100, and J23114, which have different strengths, as listed on the iGEM website (http://parts.iGEM.org/, accessed on March 13, 2023), to regulate the expression of EsaI and LasI with the goal to optimize the QS system (Fig. 1).

**Fig. 2:**
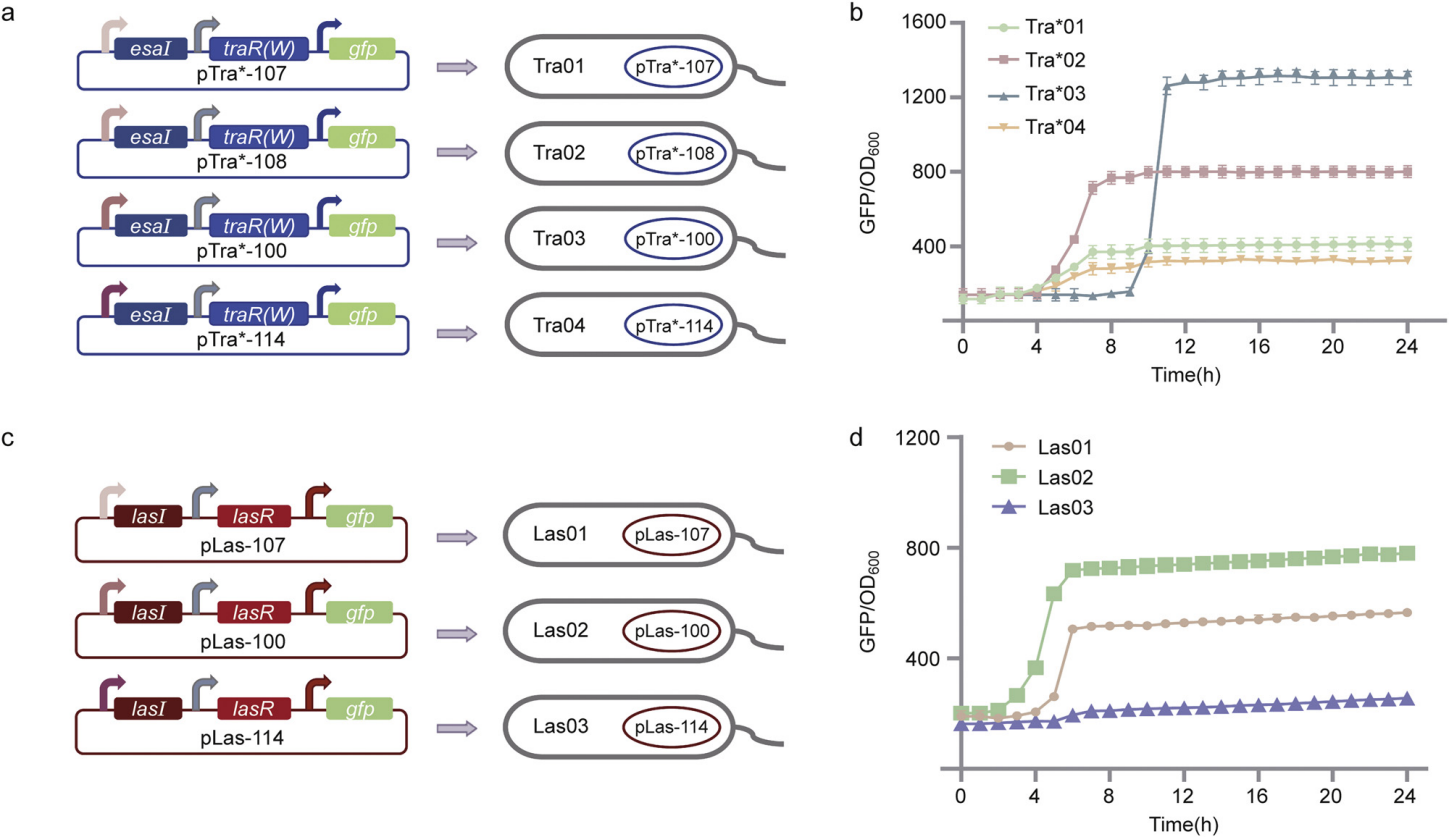
Design optimization of quorum sensing (QS) systems. (A) The optimized design of the Tra* QS system involved utilizing promoters of varying strengths to express EsaI, while the P_tra*_ promoter was employed to express green fluorescent protein (GFP). (B) Changes in green fluorescence levels in various strains with the optimized Tra* QS system were monitored over a 24-h culture period in 24-well plates. (C) The optimized design of the Las QS system utilized promoters of different strengths to express LasI, with the P_las_ promoter used for GFP expression. (D) Changes in green fluorescence levels in various strains with the optimized Las QS system were monitored over a 24-h culture period in 24-well plates. Error bars represent data from three independent biological replicates.

In the Tra* QS system, we utilized the aforementioned constitutive promoters of varying strengths to express EsaI, the constitutive promoter J23104 to express TraR(W), and P_tra*_ to induce GFP expression. The plasmids pTra*−107, pTra*−108, pTra*−100, and pTra*−114 were constructed and transformed into *E. coli* TOP10 cells for characterization (Fig. 2A). By measuring the relative fluorescence intensity of GFP in individual cells, we observed that when the Tra* QS system was activated, strains Tra01 and Tra04 initially triggered P_tra*_, resulting in GFP expression starting at 4 h, albeit at a relatively low intensity. The Tra02 strain began to express GFP at 4.5 h, with an expression intensity twice that of the Tra01 strain. In contrast, Tra03 strain initiated GFP expression at 9.5 h, exhibiting the highest expression intensity among the four groups (Fig. 2B). These results demonstrate that by regulating the expression intensity of EsaI, we successfully obtained a Tra* QS system with different expression time intervals.

For the Las QS system, we employed constitutive promoters of varying strengths to express LasI, the constitutive promoter J23104 was utilized for LasR expression, and P_las_ was used to induce GFP expression. Following the construction of the plasmids pLas-107, pLas-100, and pLas-114, they were transformed into *E. coli* TOP10 cells, leading to the establishment of the Las01, Las02, and Las03 strains, respectively (Fig. 2C). Strain characterization indicated that Las02 initiated GFP expression at 3 h and exhibited the highest expression intensity among the three groups (Fig. 2D). In contrast, strains Las01 and Las03 began expressing GFP at 4 h and showed lower fluorescent intensities than Las02. These findings suggest that utilizing promoters of different strengths enables optimization of the Las QS system to activate expression-related genes at distinct time points.

After finely regulating the AHL synthase expression level, which generates signaling molecules, a noticeable gap emerged in the activation timing of the Las and Tra* QS systems. Furthermore, genes regulated by these two systems exhibited varying expression levels. These findings demonstrate that an optimized QS system facilitates the dynamic expression of target genes at different response time intervals, thereby offering a viable option for the development of a multimodule self-induced regulatory system.

### 3.2 Screening of stationary-phase sensing systems

**Fig. 3:**
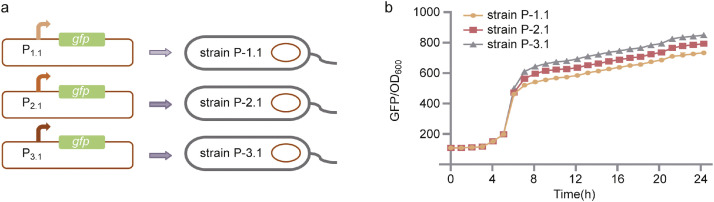
Characterization of stationary-phase sensing systems. (A) Schematic diagram of three stationary-phase sensing systems. (B) Characterization results of three stationary-phase sensing systems. Fluorescent levels were standardized using the OD_600_ values. Error bars represent data from three independent biological replicates.

A stationary-phase sensing system is a cell density-dependent expression system primarily composed of stationary phase-specific promoters [37]. These promoters are inducible elements that activate gene expression in response to bacterial growth without the need for external inducers [38]. Stationary phase-specific promoters have extensive applications in the fields of metabolic engineering and synthetic biology [39]. To select appropriate stationary phase-specific promoters for constructing a three-module regulatory system, we selected three stationary phase-specific promoters, P_1.1_, P_2.1_, and P_3.1_, which were previously reported to function in *E. coli* [39]. These promoters were used to induce the expression of the reporter gene *gfp*, enabling verification of their expression levels. Plasmids pP-1.1, pP-2.1, and pP-3.1 were constructed and introduced into *E. coli* TOP10 cells, resulting in strains P-1.1, P-2.1, and P-3.1, respectively (Fig. 3A). The characterization results indicated that the cell densities in response to the three systems were broadly consistent in *E. coli* TOP10 cells, with all systems initiating reporter gene expression at 5.5 h. Notably, the induction intensity of the P_3.1_ promoter was the highest, maintaining a consistently higher expression level throughout the experiment than that of the other two promoters. In contrast, the P_1.1_ promoter exhibited the lowest strength (Fig. 3B). These findings enabled us to obtain stationary-phase sensing systems with varying expression strengths.

### 3.3 Construction of a pathway-independent three-module self-induced system

**Fig. 4:**
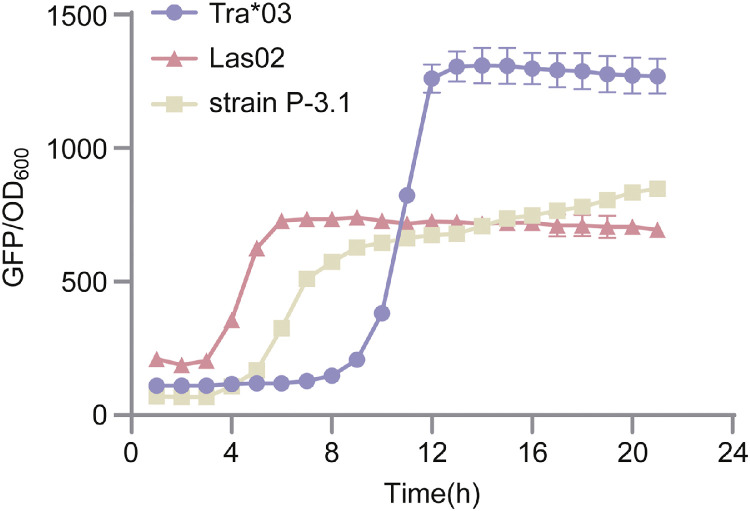
Characterization of a three-module self-induced regulatory system. Changes in green fluorescence in various strains were monitored over a 24-h culture period in 24-well plates. Fluorescent levels were standardized using the OD_600_ values. Error bars represent data from three independent biological replicates.

Given that the expression time of the stationary-phase induction system was consistent, we selected the P_3.1_ promoter, which exhibited the highest expression intensity, as a component of the three-module self-induced regulatory system to control the expression of the target gene. After analyzing the optimized QS system, we selected the Las and Tra* systems, which regulate AHL synthase expression via the promoter J23100. We integrated these systems with a stationary-phase sensing system to construct a three-module regulatory system characterized by an expression time interval and self-induction capability. To experimentally validate the temporality of the modules, we characterized each module individually using three separate reporter strains, each containing only one of the three modules, and expressed a GFP reporter under the control of the corresponding output promoter. Within this three-module regulatory system, gene expression was initiated by bacterial cell growth. Genes regulated by the Las QS system commenced expression as early as 3 h, followed by those regulated by the stationary phase induction system at 5 h; finally, the Tra* system began expression at 9.5 h. In summary, we constructed a pathway-independent three-module regulatory system that autonomously activated the expression of distinct genes at different time intervals (Fig. 4).

### 3.4 Application in redistributing carbon flux during 3-HP biosynthesis

**Fig. 5:**
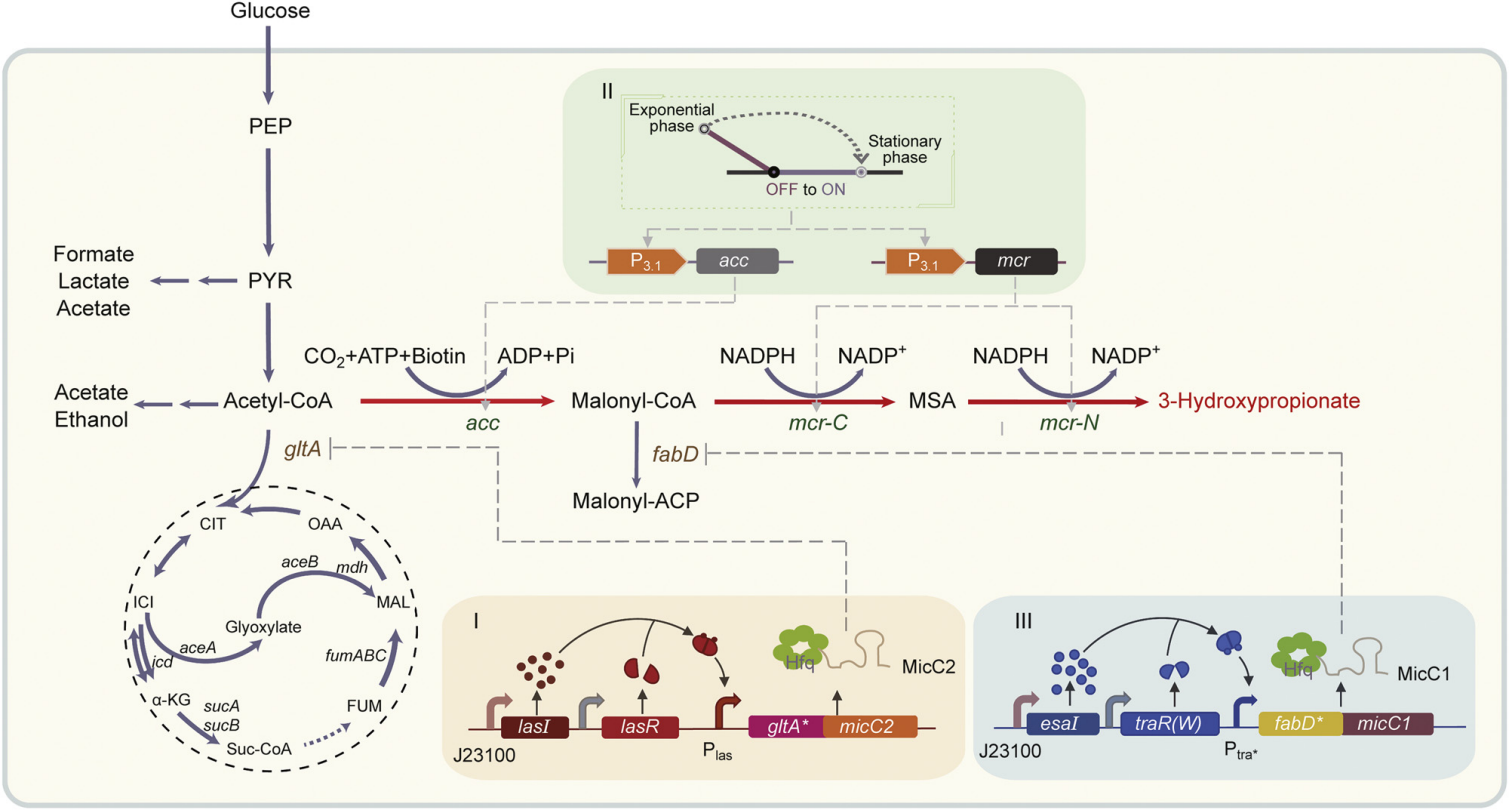
Schematic diagram of the production pathway of 3-hydroxypropionic acid (3-HP) and its three-module self-induced regulation. (I) The first regulatory module inhibits *gltA*, leading to the accumulation of acetyl-CoA. (II) The second regulatory module targets the exogenous genes synthesizing 3-HP, *acc* and *mcr*. (III) The third regulatory module suppresses the expression of *fabD*, thereby promoting the accumulation of the precursor malonyl-CoA. In this diagram, green color represents exogenous genes, brown color indicates gene with inhibited expression, and red arrows denote enhanced metabolic pathway. gltA* refers to the 24-bp sequence that serves as the target binding site in *gltA*, while fabD* denotes the 24-bp sequence functioning as the target binding site in *fabD. micC1* and *micC2* represent two backbones of artificial trans-encoding small RNAs.

The compound 3-HP is a promising value-added chemical [[40], [41], [42]]. Metabolic engineering has been conducted in various microorganisms, including *E. coli, Klebsiella pneumoniae*, and yeast, resulting in the development of different chassis cells for 3-HP production [43,44]. In *E. coli*, 3-HP is primarily synthesized via the malonyl-CoA pathway, utilizing glucose as a precursor [45]. Within this pathway, acetyl-CoA is converted to malonyl-CoA by acetyl-CoA carboxylase (Acc), which is subsequently catalyzed by the NADPH-dependent malonyl-CoA reductase (MCR) in a two-step reaction that produces 3-HP [43]. Because the tricarboxylic acid (TCA) cycle and fatty acid metabolism consume the precursor substances acetyl-CoA and malonyl-CoA, it is crucial to regulate these pathways to enhance 3-HP synthesis without compromising cell growth. In the present study, we implemented a three-module regulatory system for 3-HP production (Fig. 5).

The first regulatory module was designed as follows. First, we utilized an optimized Las QS system to dynamically regulate the expression of artificial trans-encoding small RNA, thereby inhibiting the expression of acetyl-CoA encoded by the *gltA* gene. Specifically, when a signaling molecule reaches a certain threshold, it activates P_las_, which in turn leads to the expression of MicC2, further inhibiting the translation of the *gltA* gene. This process redirects metabolic flow from the TCA cycle to the production of 3-HP. The second regulatory module was designed to decouple cell growth from product synthesis by employing a stationary phase-specific promoter to control the temporal expression of the synthetic pathway genes *acc* and *mcr* in *E. coli*. The third regulatory module was designed based on the intermediate product malonyl-CoA, which is encoded by *fabD*. Static blockade of malonyl-CoA is detrimental to cell growth, as it is a fundamental building block of fatty acid biosynthesis [46,47]. To address this, we employed the Tra* QS system to regulate the expression of artificial trans-encoding small RNA, thereby dynamically inhibiting the translation of *fabD* and leading to the accumulation of malonyl-CoA for the enhanced production of 3-HP.

**Fig. 6:**
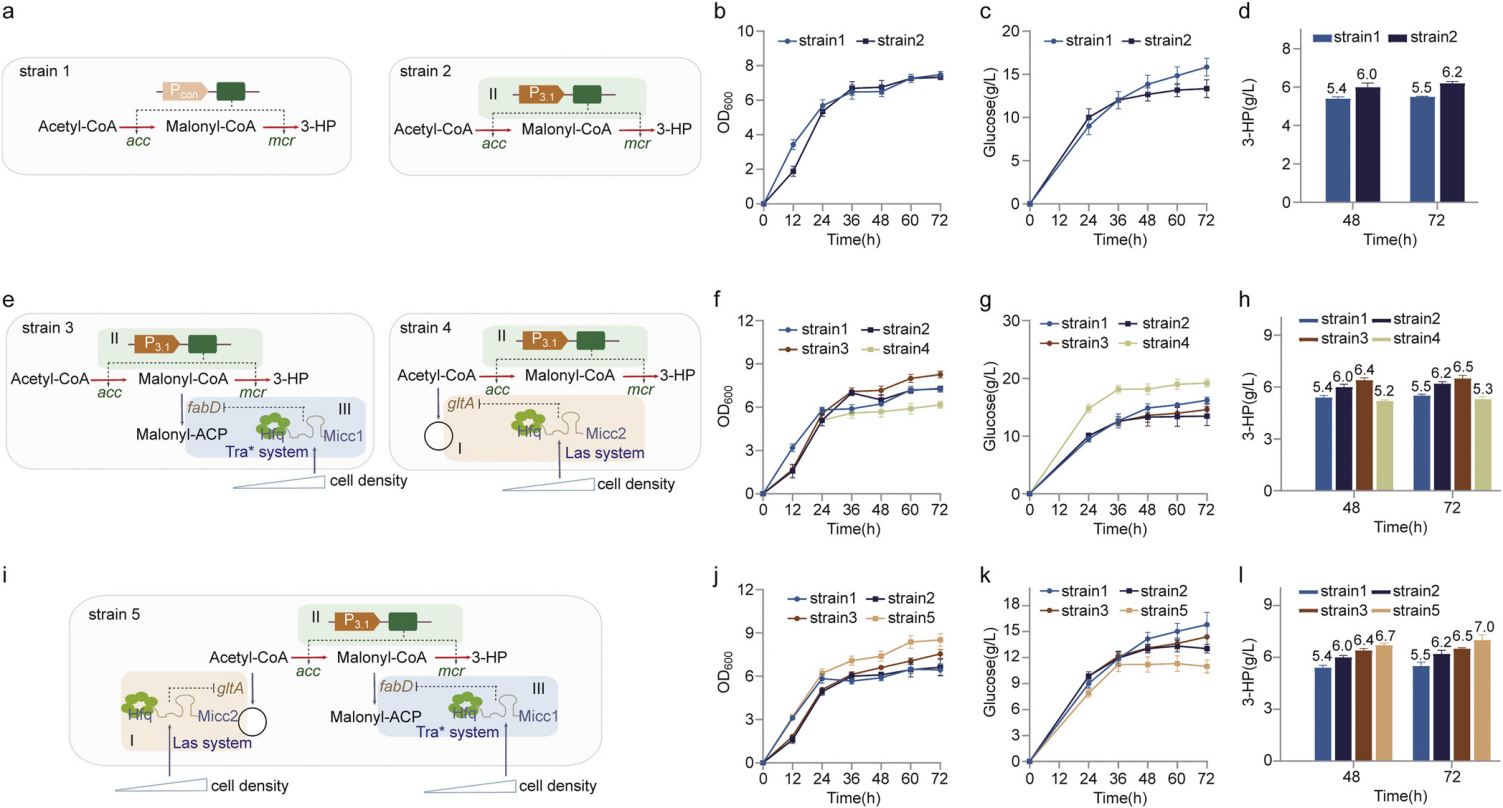
Construction of a 3-hydroxypropionic acid (3-HP) production strain and fermentation results. (A) Schematic diagram of the construction of static regulatory strain 1 and single-module regulatory strain 2. P_con_ represents a constitutive promoter. (B–D) Growth, glucose consumption, and titer of strains 1 and 2. (E) Schematic diagram of the construction of double-module regulatory of strains 3 and 4. (F–H) Growth, glucose consumption, and titer of strains 1–4. (I) Schematic diagram of the construction of three-module regulatory strain 5. (J–L) Growth, glucose consumption, and titer of production strains. All results were obtained from three independent biological replicates (n = 3).

We utilized *E. coli* TOP10 as the starting strain and implemented a three-module regulatory system to facilitate 3-HP production. Acc derived from *Corynebacterium glutamicum* and MCR derived from *C. aurantiacus* were introduced to establish production lines [48]. Acc and MCR were constitutively expressed using high- and low-copy plasmids, respectively, leading to the construction of a static regulatory strain, referred to as strain 1, for 3-HP production (Fig. 6A).

In this study, we focused on validating the effect of modular stacking on the overall performance of the engineered strains; specifically, the progressive changes in growth, substrate consumption, and target product accumulation as the regulatory modules were increased stepwise from a single to three modules. Accordingly, we used a static constitutive expression strain (strain 1) as the control and sequentially stacked the regulatory modules to construct strains 2–5, allowing us to observe the performance gains achieved by adding the regulatory modules layer-by-layer. Initially, strain 2 was constructed for 3-HP production using only the second regulatory module (Fig. 6A). Following successful construction of the single-module regulatory strain, shake flask fermentation was conducted. The fermentation results indicated that the growth curves and glucose consumption of the static regulatory strain (strain 1) and single-module regulatory strain (strain 2) were similar (Fig. 6B–C). The titer of 3-HP in strain 2 increased to 6.2 g/L compared with 5.5 g/L in strain 1 (Fig. 6D). These results indicate that the dynamic regulation in the expression of 3-HP synthesis genes is conducive to product accumulation.

In the second step, we constructed two-module regulatory strains, strains 3 and 4 (Fig. 6E). After strain construction, shake-flask fermentation was conducted using static and self-induced regulatory strains. The results indicated that after 72 h of fermentation, the titer of strain 3 increased by 18% compared with that of strain 1 (Fig. 6H); a noticeable improvement in growth was observed (Fig. 6F); glucose consumption did not differ significantly between the groups (Fig. 6G). However, despite increased glucose consumption, both the growth and titer of strain 4 declined, indicating that the dual-module regulation did not produce the intended effect. This further reflects the complexity of metabolic regulatory networks. Our experimental results (Fig. 6F) showed a clear growth difference between strains 2 and 3, with strain 3 carrying an additional *fabD* repression module compared with strain 2. Both strains exhibited similar growth during the early exponential phase, whereas strain 3 exhibited significantly better growth than strain 2 during the late exponential and stationary phases. These results confirmed that the moderate *fabD* repression strategy employed in this study did not cause irreversible damage to the cells. However, inappropriate regulation of the strength or timing of repression may compromise cell viability.

Subsequently, we employed a three-module self-inducing system to construct a production strain designated as strain 5 (Fig. 6I). During this regulatory process, as the strain grows, the first regulatory module is activated, wherein the Las QS system inhibits *gltA* and accumulates acetyl-CoA. Subsequently, the second regulatory module initiates the expression of *acc* and *mcr*, thereby accelerating the conversion of acetyl-CoA to 3-HP. Finally, the activation of the third regulatory module can inhibit *fabD* expression via the Tra* QS system, thereby reducing malonyl-CoA flow to the competitive pathway [49,50].

The results of shake-flask fermentation indicated that the 3-HP titer of the three-module regulatory strain increased to 7.0 g/L (Fig. 6L), representing increases of 8%, 13%, and 27% compared to the double-, single-, and static regulatory strains, respectively. Additionally, strain 5 exhibited the highest final OD_600_ (Fig. 6J), with lower glucose consumption than the other strains (Fig. 6K). These experimental results demonstrate that the three-module self-induced system outperformed the single- and double-module systems for 3-HP biosynthesis, offering advantages in promoting strain growth and enhancing titers. Three-module self-induced regulation typically provides better growth, less carbon consumption, and a higher titer; however, the improvements presented here for 3-HP are incremental.

## 4 Conclusion

In this study, we developed a three-module self-inducing system whose design principle was based on cellular physiological state signals rather than on specific pathway metabolites. Consequently, the system can be adapted to other metabolic contexts by simply replacing the target genes with the corresponding small RNA sequences. By coupling each regulatory module to the cell density and growth phases, the system achieves temporal coordination among multiple regulatory modules. We applied this system for the biosynthesis of 3-HP and achieved a titer of 7.0 g/L during shake-flask fermentation. In summary, this system offers a modular regulatory platform for balancing cell growth and product synthesis and holds promise for addressing key challenges in biosynthesis, such as unbalanced resource allocation, overexpression of heterologous genes, and excessive metabolic burden. The three-module self-induced system developed in this study can serve as an effective tool for modulating the microbial metabolic status and enhancing the production efficiency of microbial cell factories.

## Data availability statement

The data supporting the findings of this study are included in the published article as well as in the Supplementary Materials available online.

## CRediT authorship contribution statement

**Yaping Gao:** Writing – original draft, Investigation, Conceptualization. **Wendi Xu:** Formal analysis, Data curation. **Xiaoya Yang:** Formal analysis, Data curation. **Fei Gu:** Formal analysis, Data curation. **Sumeng Wang:** Formal analysis, Data curation. **Qingsheng Qi:** Writing – review & editing, Supervision, Project administration, Funding acquisition, Conceptualization. **Quanfeng Liang:** Writing – review & editing, Supervision, Project administration, Funding acquisition, Conceptualization.

## Declaration of competing interest

The authors declare that they have no known competing financial interests or personal relationships that could have appeared to influence the work reported in this paper.

## Appendix Supplementary materials

Supporting information for the strains, plasmids, promoters, and sequences used in this study is available free of charge. Image, application 1
