# Supplementary material for: Construction of a pathway-independent three-module self-induced regulatory system and its application in 3-hydroxypropionic acid production
Source: Eng Microbiol. 2026 Jun 5;6(3):100281. doi: 10.1016/j.engmic.2026.100281 (PMC13329355; doi:10.1016/j.engmic.2026.100281)
Supplement: Supplementary file 1 — Supporting information for the strains, plasmids, promoters, and sequences used in this study is available free of charge. [file mmc1.docx]

**Construction of a pathway-independent three-module self-induced regulatory system and its application in 3-hydroxypropionic acid production**

Yaping Gao^a^, Wendi Xu^a^, Xiaoya Yang^a^, Fei Gu^a^, Sumeng Wang^b^, Qingsheng Qi^a^, Quanfeng Liang^a*^

^a^State Key Laboratory of Microbial Technology, Shandong University, Qingdao, 266000, PR China

^b^Qingdao Agricultural University, Qingdao, 266100, China.

*Corresponding author.

E-mail addresses: [liangquanfeng@sdu.edu.cn](mailto:liangquanfeng@sdu.edu.cn) (Q. Liang).

| **Supplementary Table S1.** Strains used in this study. | | |
| --- | --- | --- |
| **Strains** | **Relevant characteristics** | **References** |
| DH5α | *F^–^ endA1 glnV44 thi-1 recA1 relA1 gyrA96 deoRnupG purB20 φ80dlacZ∆M15 ∆(lacZYA-argF) U169, hsdR17(rK^–^mK^+^), λ^–^* | Lab stock |
| *E. coli* TOP10 | *F^-^ mcrAΔ(mrr-hsdRMS-mcrBC) φ80lacZΔM15 ΔlacX74nupG recA1 araD139Δ(ara-leu)7697 galE15 galK16 rpsL(StrR) endA1 λ^-^* | Lab stock |
| Tra01 | *E. coli* TOP10 harboring pTra*-107 | This study |
| Tra02 | *E. coli* TOP10 harboring pTra*-108 | This study |
| Tra03 | *E. coli* TOP10 harboring pTra*-100 | This study |
| Tra04 | *E. coli* TOP10 harboring pTra*-114 | This study |
| Las01 | *E. coli* TOP10 harboring pLas-107 | This study |
| Las02 | *E. coli* TOP10 harboring pLas-100 | This study |
| Las03 | *E. coli* TOP10 harboring pLas-114 | This study |
| strain P-1.1 | *E. coli* TOP10 harboring pP-1.1 | This study |
| strain P-2.1 | *E. coli* TOP10 harboring pP-2.1 | This study |
| strain P-3.1 | *E. coli* TOP10 harboring pP-3.1 | This study |
| Strain 1 | *E. coli* TOP10 harboring pH-I and pL-I | This study |
| Strain 2 | *E. coli* TOP10 harboring pH-II and pL-II | This study |
| Strain 3 | *E. coli* TOP10 harboring pH-III and pL-II | This study |
| Strain 4 | *E. coli* TOP10 harboring pL-III and pH-II | This study |
| Strain 5 | *E. coli* TOP10 harboring pH-III and pL-III | This study |

| **Supplementary Table S2.** Plasmids used in this study. | | |
| --- | --- | --- |
| **Plasmids** | **Relevant characteristics** | **References** |
| pTra*-107 | Cm^R^, BBa_J23107-RBS 30-*esaI*, BBa_J23104-RBS30-*traR(W)*, P_tra*_-*gfp*, ori pBR322 | This study |
| pTra*-108 | Cm^R^, BBa_J23108-RBS 30-*esaI*, BBa_J23104-RBS30-*traR(W)*, P_tra*_-*gfp*, ori pBR322 | This study |
| pTra*-100 | Cm^R^, BBa_J23100-RBS30-*esaI*, BBa_J23104-RBS30-*traR(W)*, P_tra*_-*gfp*, ori pBR322 | This study |
| pTra*-114 | Cm^R^, BBa_J23114-RBS30-*esaI*, BBa_J23104-RBS30-*traR(W)*, P_tra*_-*gfp*, ori pBR322 | This study |
| pLas-107 | Amp^R^, BBa_J23107-RBS30-*lasI*, BBa_J23104-RBS30-*lasR*, P_las_-*gfp*, ori p15A | This study |
| pLas-100 | Amp^R^, BBa_J23100-RBS30-*lasI*, BBa_J23104-RBS30-*lasR*, P_las_-*gfp*, ori p15A | This study |
| pLas-114 | Amp^R^, BBa_J23114-RBS30-*lasI*, BBa_J23104-RBS30-*lasR*, P_las_-*gfp*, ori p15A | This study |
| pP-1.1 | Cm^R^, P_1.1_-*gfp*, ori p15A | This study |
| pP-2.1 | Cm^R^, P_2.1_-*gfp*, ori p15A | This study |
| pP-3.1 | Cm^R^, P_3.1_-*gfp*, ori p15A | This study |
| PH-I | Cm^R^, BBa_J23107-RBS30-*acc*, ori pBR322 | This study |
| pL-I | Amp^R^, BBa_J23104-RBS30-*mcr*, ori p15A | This study |
| pH-II | Cm^R^, P_3.1_-RBS30-*acc*, ori pBR322 | This study |
| pL-II | Amp^R^, P_3.1_-RBS30-*mcr*, ori p15A | This study |
| pH-III | Cm^R^, P_3.1_-RBS30-*acc*, BBa_J23100-RBS30-*esaI*, BBa_J23104-RBS30-*traR(W)*, Pt_ra*_-MicC1-fabD, ori pBR322 | This study |
| PL-III | Amp^R^, P_3.1_-RBS30-*mcr*, BBa_J23100-RBS30-*lasI*, BBa_J23104-RBS30-*lasR*, P_las_-MicC2-gltA, ori p15A | This study |

| **Supplementary Table S3.** Primers used in this study. | |
| --- | --- |
| Name | Sequence |
| 107-ESAI-F | CCCTAGGTATTATGCTAGCTCTAGAGTCACACAGGACTACTAGATGGATGCTGGAGCTGTTCGACG |
| 107-ESAI-R | GTGACTCTAGAGCTAGCATAATACCTAGGGCTGAGCTAGCCGTAAACATGAGCAGATCCTCTACGCC |
| 108-ESAI-F | TCCTAGGTATAATGCTAGCTCTAGAGTCACACAGGACTACTAGATGGATGCTGGAGCTGTTCGACG |
| 108-ESAI-R | TCCTAGGTATAATGCTAGCTCTAGAGTCACACAGGACTACTAGATGGATGCTGGAGCTGTTCGACG |
| Tra-F | CCTAGGTACAGTGCTAGCTCTAGAGAAAGAGGAGAAATACTAGATGGATGCTGGAGCTGTTCGAC |
| Tra-R | TCTTTCTCTAGAGCTAGCACTGTACCTAGGACTGAGCTAGCCGTCAACATGAGCAGATCCTCTACGCC |
| 114-ESAI-F | TCCTAGGTACAATGCTAGCTCTAGAGTCACACAGGACTACTAGATGGATGCTGGAGCTGTTCGACG |
| 114-ESAI-R | GTGACTCTAGAGCTAGCATTGTACCTAGGACTGAGCTAGCCATAAACATGAGCAGATCCTCTACGCC |
| 107-LASI-F | GTGACTCTAGAGCTAGCATAATACCTAGGGCTGAGCTAGCCGTAAACACGAATTCGAAATGTGCGCTT |
| 107-LASI-R | CCCTAGGTATTATGCTAGCTCTAGAGTCACACAGGACTACTAGATGGATGATCGTACAAATTGGTCGGCG |
| Las-F | TCTTTCTCTAGAGCTAGCACTGTACCTAGGACTGAGCTAGCCGTCAACACGAATTCGAAATGTGCGCTTC |
| Las-R | CCTAGGTACAGTGCTAGCTCTAGAGAAAGAGGAGAAATACTAGATGGATGATCGTACAAATTGGTCGGCG |
| 114-LASI-F | GTGACTCTAGAGCTAGCATTGTACCTAGGACTGAGCTAGCCATAAACACGAATTCGAAATGTGCGCTTC |
| 114-LASI-R | TCCTAGGTACAATGCTAGCTCTAGAGTCACACAGGACTACTAGATGGATGATCGTACAAATTGGTCGGCG |
| P3-F | CTCCCTTATGCGACTCCTGCATTAGGAAATGACGGTCGGGTAACTACCAC |
| P3-R | CTAGTATTTCTCCTCTTTCT |
| 107-ACC-F | CCGCTTACAGACAAGCACTGCGACTCCTGCATTAG |
| 107-ACC-R | GCAGGAGTCGCAGTGCTTGTCTGTAAGCGGATGCC |
| 104-MCR-F | CACCTGACGTCTAAGCACGAATTCGAAATGTGCGC |
| 104-MCR-R | CATTTCGAATTCGTGCTTAGACGTCAGGTGGCACC |
| 3.1-ACC-F | GACAAGCTGTGACCGGACGGTCGGGTAACTACCAC |
| 3.1-ACC-R | AGTTACCCGACCGTCCGGTCACAGCTTGTCTGTAAG |
| MCR-3.1-F | CACCTGACGTCTAAGGAATTCGAAATGTGCGCCGAC |
| MCR-3.1-R | GCACATTTCGAATTCCTTAGACGTCAGGTGGCACC |
| Las-gltA-F | ACCGAAATCTATCTCATTTGCTAGTTATAAAATTATGAAATTTGCGTAAATTCTTCAGGGTGAGTTTTGCTTTTGTATCAGCC |
| Las-gltA-R | TTATAACTAGCAAATGAGATAGATTTCGGTGAACCCGGACCCTTGCTAGGCTCGAACTTAGACGTCAGGTGGCACC |
| Las-gltA-1-F | CTTTTCTCTTCTCAAGGCGTATCACGAGGCAGAATTTC |
| Las-gltA-1-R | GCCTCGTGATACGCCTTGAGAAGAGAAAAGAAAACCGCCG |
| Micc-fabd-F | ACTGATTTTCCAACATATAAAAAGACAACATCCCGACCCCCTCAGGGTCGGGATTTTTTTTGGAAACACAGAAA |
| Micc-fabd-R | GGCAATGCAATGGCCCAACAGAAACGCGCTGGTTCGTTTCTAGGATAAGGATATTCGACTATAACAA |
| Tra-fabd-F | TTTTTCGACCAAAGGCCGAAACAAGCGCTCATGAG |
| Tra-fabd-R | GAGCGCTTGTTTCGGCCTTTGGTCGAAAAAAAAAGCCCG |

**Supplementary Table S4.** Promoters used in this study.

| **Promoters** | **Sesquences(5’-3’)** | |  |
| --- | --- | --- | --- |
| Plas | TTCGAGCCTAGCAAGGGTCCGGGTTCACCGAAATCTATCTCATTT GCTAGTTATAAAATTATGAAATTTGCGTAAATTCTTCA | |  |
| Ptra* | GCACGTGCAGATCTGCACATTTACGCAAGAAAATGGTTTGTTATA GTCGAATAT | |  |
| BBa_J23104 | TTGACAGCTAGCTCAGTCCTAGGTATTGTGCTAGC | |  |
| BBa_J23107 | tttacggctagctcagccctaggtattatgctagc | |  |
| BBa_J23108 | CTGACAGCTAGCTCAGTCCTAGGTATAATGCTAGC | |  |
| BBa_J23100 | ttgacggctagctcagtcctaggtacagtgctagc | |  |
| BBa_J23114 | tttatggctagctcagtcctaggtacaatgctagc | |  |
| P_1.1_ | GacggtcgcggtaactacacacttggtatcaaagaAcagTtaatcttccctgatattgactacaataaagTaacaaaggTtcgcggaatggacatcgttatcgtaAcaactgctaataCtgacgaagaagctcgtgagctAttaactcaagtactgatGccgttccagaaataatCaatgaaagggaggcgaaatcgtggctaaaaagtctatgattgcgaaacaacaacgt | |  |
| P_2.1_ | gacggtcgcggtaactacacacttggtatcaaagaacagttaatcttccctgatattgactacaataaagtaacaaaggttcgcggaatggacatcgttatcgtaacaactgctaatactgacgaagaagctcgtgagctattaactcaagtagtgatgccgttccagaaataatcaatgaaagggaggcgaaatcgtggctaaaaagtctatgattgcgaaacaacaacgt | |  |
| P_3.1_ | gacggtcgggtaactaccacttggtatcaaagaacagttaatcttcccagatattgactacaataaagtaacaaaggttcgcggaatagacatcgttatcgtaacaactgctaatactgacgaagaagctcgtgagctattaactcaagtagtgatgccgttccagaaataatcaatgaaagggaggcgaaatcgtggctaaaaagtctatgattgcgaaacaacaacgt | |  |
| **Supplementary Table S5.** Sequences used in this study. | | | |
| Name | | Sequences | |
| P_tra*_-*fabD**-MicC1 | | GCACGTGCAGATCTGCACATTTACGCAAGAAAATGGTTTGTTATAGTCGAATATgggaacacaaatgcaaattgcgtcTTTCTGTTGGGCCATTGCATTGCCACTGATTTTCCAACATATAAAAAGACAAGCCCGAACAGTCGTCCGGGCTTTTTTTttcagccaaaaaacttaagaccgccggtcttgtccactaccttgcagtaatgcggtggacaggatcggcggttttcttttctcttctcaa | |
| P_las_-*gltA*-*MicC2 | | TTCGAGCCTAGCAAGGGTCCGGGTTCACCGAAATCTATCTCATTTGCTAGTTATAAAATTATGAAATTTGCGTAAATTCTTCAggctgatacaaaagcaaaactcacTTTCTGTTGGGCCATTGCATTGCCACTGATTTTCCAACATATAAAAAGACAACATCCCGACCCCCTCAGGGTCGGGATTTTTTTTggaaacacagaaaaaagcccgcacctgacagtgcgggctttttttttcgaccaaagg | |

The atsRNA is expressed under the control of the P_las_ promoter or P_tra*_ promoter (yellow sequence). The atsRNA target-binding sequence is inserted into the red region, which is *fabD** or *gltA** have 24 nucleotides long. The Hfq-recognizing stem-loop structure is marked in blue. The interspace sequence and poly-T tail of MicC were kept intact to maintain sRNA function, while the inner terminator of MicC was switched to those of other sRNA structures, which are marked in green, to avoid homologous recombination. The downstream additional terminators shown in lower case were precharacterized as nonhomologous with each other [1].

**References**

[1] D. Na, S.M. Yoo, H. Chung, H. Park, J.H. Park, S.Y. Lee, Metabolic engineering of Escherichia coli using synthetic small regulatory RNAs, Nat Biotechnol 31 (2013) 170-174.
